# Comprehensive Women Health Services at Beverage-Producing Industries of Limpopo (South Africa): Women’s Perspective

**DOI:** 10.3390/ijerph17228293

**Published:** 2020-11-10

**Authors:** Livhuwani Muthelo, Masenyani Oupa Mbombi, Mamare Adelaide Bopape, Tebogo Maria Mothiba

**Affiliations:** 1 Department of Nursing Science, University of Limpopo, Mankweng 0727, South Africa; masenyani.mbombi@ul.ac.za (M.O.M.); mamare.bopape@ul.ac.za (M.A.B.); 2 Faculty of Health Sciences Dean’s Office, University of Limpopo, Mankweng 0727, South Africa; tebogo.mothiba@ul.ac.za

**Keywords:** experiences, women health care services, food manufacturing industries, healthcare hazards, comprehensive women health services

## Abstract

(1) Background: Women remain highly vulnerable to numerous risks at work, including labor rights violations, violence and harassment, myriad general and reproductive health risks. The availability of the comprehensive services remains the only hope for these women, yet very little is known about their perspective. (2) Aim: To determine the experiences of women regarding the availability of comprehensive women’s health services in the industries of Limpopo (South Africa). (3) Methods: The project adopted the qualitative research method to determine the experiences of women related to the availability of comprehensive women’s health services. Non-probability purposive and convenience sampling was used to select 40 women employed in two beverage producing industries. A semi-structured interview with an interview guide was used to collect data that were analyzed using thematic analysis. (4) Results: Four themes emerged about the available health services in the two industries; diverse experiences related to available women’s health services, knowledge related to women’s health services, and diverse description of women’s health services practice and risks. The themes are interpreted into ten sub-themes. (5) Conclusions and Recommendations: There is a lack of available comprehensive women health services at the two beverage producing industries. Thus, women face challenges regarding accessing comprehensive women’s reproductive health care services as well as being exposed to health hazards such as burns, bumps, injuries and suffering from inhalation injuries and burns from moving machines, noise, slippery floors, and chemicals that are used for production in the industry. Women expressed dissatisfaction in the industries regarding the provided general health and primary healthcare services that have limited women’s health-specific services. We recommended that the industries should prioritize designing and developing the comprehensive women health services that to enable women at the industries to have access to good-quality reproductive health care and effective interventions.

## 1. Introduction

The World Health Organization [WHO] has documented a global diverse experiences of realities in industry and in the community or at home for both men and women [1]. Furthermore, men and women also encounter different experiences of health effects due to their biological and social differences in industry [2]. Given these differences, the authors question if there are comprehensive health services for women in industry to cater for the detrimental health effects. In 2007 the WHO recommended the Global Plan of Action for Workers Health which includes all aspects such as the prevention, promotion, protection, and maintenance of workers’ health from potential health problems caused by the working conditions [3]. However, Rantanen et al. [4] identified a gap of lower coverage of employees’ health care services in developing regions. Thus, providing a universal need to increase the coverage of workers by occupational health services throughout the world [4].

Globally there has been a fundamental change of gender make-up in the industries with more women into the workforce [5,6]. Although the change is significant, there still a gap with the industries health policies and practices that are not keeping up with the changes in order to accommodate the influx of women health issues within the industries [7]. Pearce [7] also agrees that the industries’ health policies still accommodate the traditional occupational health and safety compliance which was developed decades ago with men being dominant workforce while not accommodating the health challenges of women [8]. Wofford, et al. [5] argued that though an increase in the acknowledgment of the regulation of women’s health and safety which includes protections against pregnancy and breastfeeding mothers, and restrictions of exposure to chemicals that harm reproductive health, there is still a gap on how industries should be gender-equitable and ensure women’s access to general and reproductive health services and products. The aforementioned authors also argued that there is a lack of standards that should apply to industries health facilities and providers, and standards of directing how industries policies should address the specific health needs and industries hazards that represent a particular risk for women, or how occupational health and public health systems should connect. In South Africa, there are several laws and improvements in regulations for ensuring the protection of women in the industries as outlined below [9]:

- Employment Equity Act 55 of 1998: Protects workers from unfair discrimination including gender, sex, pregnancy, marital status, and family responsibility.
- Basic Conditions of Employment Act 75 of 1997: Gives effect to the right to fair labor practices and maternity leave protection. Women have the right to four months of maternity leave and further stipulate that employees have a right to at least four consecutive months’ unpaid maternity.
- Equal pay protection: A code of good practice on equal pay for work of equal value states that the equal pay principle addresses a specific aspect of industries discrimination which includes gender and sex.

Given the availability and improvement in the regulations to protect women’s health, the question on the availability of comprehensive health services in the industries to promote, protect, prevent, and maintain women’s health remains unanswered. Hence the purpose of the current project which focuses on the availability of comprehensive health services for women working in beverage producing industries. For this project, an industry shall refer to the working area where women are involved in the production and manufacturing of beverages in Limpopo Province. This project was done in two private beverage producing industries that operate within a Corporate Social Responsibility (CSR) System. Corporate Social Responsibility (CSR) is a self-regulating business model that helps a company be socially accountable to itself, its stakeholders, and the public [10]. Wofford et al. [5] reported that CSR has policies that focus more on shaping corporate procedures and practices related to the environment, labor, and human rights, however, these policies seem to be ignoring women’s health needs within the industries. Considering that women have many exceptional health concerns, which range from menstrual cycles, pregnancy, birth control, menopause, and musculoskeletal disorders, it is a concern that the CSR policies still have overlooked women’s health issues [5]. On the other hand, women are more often caregivers who perform the bulk of housework and care for children, which increases the risk of musculoskeletal disorders and psychological problems. For instance, Swanson, et al. [11] noted that balancing work and family tasks put additional stress on women, who in many families still take primary responsibility for childcare. When family and work demands collide, it results in stress that affects the physical and mental health of women [11]. Whereas Dudley, et al. [12] documented that every woman of working age experiences some health-related, physical, and psychological problems such as job stress which Sawnson et al. [11] reported a link with heart disease, muscle/bone disorders, depression, and burnout amongst women. It is important to note that women with unhealthy lifestyles and chronic health conditions are less productive, take more sick leave and they tend to be frequently absent from work which pose health hazards to workforce remaining at work [13,14].

Industries’ hazards can affect reproductive health, the ability to become pregnant and the health of unborn children [15]. According to the Work Foundation Report (WHP) by Dudley et al. [12], industries hazards exposure can affect women reproductive health in various ways: Sexual functioning, menstrual health, fertility (for women and men), pregnancy, breastfeeding, certain cancers (e.g., prostate, breast, cervix), menopause, and children’s development. However, the Work Foundation Report [12], revealed unrecognized chronic gynecological, musculoskeletal and reproductive health conditions which hold back women’s productivity and could be damaging to their career progress and development within the industries.

The unrecognized chronic gynecological, musculoskeletal and reproductive health conditions might render the industries unproductive, given the fact that, Catalyst [12] indicated that despite the challenges faced by women at work, there is an anticipated increase in the number of women in male-dominated work industries. As such, women working in male-dominated industries face challenges, such as an unsafe working environment with reported cases of sexual harassment [12]. In a 2017 survey, 62% of the women interviewed who work in male-dominated industries in the United States reported that sexual harassment is a problem in their industry, compared to 46% of women working in female-dominated industries [12]. The above statistics further raises questions regarding the health of women in the industries.

According to Statistics South Africa [16], South Africa has made great strides, but gender representatives are still below the 50% mark for positions that come with a great deal of influence, and women’s health is of no exception. In South Africa, women accounted for 43.8% of total employment in the second quarter of 2018 with only 32% of managers being women. For instance, there are more women than men employed in the informal sector Trade with 47.6% of women compared to 30.6% of men [16]. Women dominated the domestic worker and Clerk or Technician occupations, with men dominating the rest. The marked increase of women in the industries necessitates the reflections on the current women’s health services provided at the industries. International Labour Organization (ILO) [17] documented that the Occupational Safety and Health (OSH) hazards affecting women workers have been traditionally under-estimated because OSH standards and exposure limits to hazardous substances were previously developed based on male populations. Based on the above background, the authors argue that there is a lack of women-specific services which has been particularly disadvantaged by out-of-date workforce structures, industries arrangements, and the current OSH standards within the industries. Therefore, the current study aims to determine the availability of comprehensive women’s health services in industries having many women.

## 2. Materials and Methods

### 2.1. Study Setting

The study was conducted at two beverage producing industries which are situated in the Polokwane Local Municipality. The Polokwane Local Municipality is located within the Capricorn District in the Limpopo Province of South Africa. The municipality serves as the economic hub for the province and has the highest population density in the Capricorn District. The Polokwane Local Municipality comprises the central business district, industrial area, and a range of social services and well-established formal urban areas servicing the more affluent residents of Polokwane. The study took place in industrial areas which has various industries for commercial purpose. For example, two beverage producing industries were the focus of the current project. The selected industries employ both men and women, making it relevant to the study. For instance, *Industry number 1* is a beverage company producing soft drinks which has 1160 employees (785 permanent and 375 contractors, with 200 females including contractors). Furthermore, the company has 12 departments, including packaging, sales and marketing and distribution and the health and safety department with an occupational health clinic onsite. The identified health hazards in *Industry 1* are presented in Table 1. *Industry 2* is also a beverage-producing company for liquor which has approximately 400 employees (with 160 females). This industry has nine departments including brewing, packaging, depot, sales and the health and safety department with an occupational health onsite clinic. The health hazards identified in *Industry 2* are presented in Table 2. Both industries have on-site occupational health clinics, which provide occupational and primary health care services to employees.

### 2.2. Research Design

According to Vaismoradi, et al. [18], qualitative research aims to arrive at an understanding of a particular phenomenon from the perspective of those experiencing it. The project adopted a qualitative explorative and descriptive designs to explore the experiences of women in industries regarding the availability of comprehensive women health services in their industries. The objective of the study was to give a narrative about the women’s experiences, feelings, understanding and opinions about the comprehensive health services in their industries. As such the explorative and descriptive designs were adopted to collect and capture data that is grounded on the experiences of women in their industries [19,20]. Moreover, this enabled the researchers to discover the new meanings and appreciations that can be developed to inform, or even re-orient the importance of comprehensive women’s health at the industries [21,22].

### 2.3. Population and Sampling

The population consisted of all women employed in two selected industries including the contractors with the occupational health nurses working on the onsite clinics. Cresswell et al. [23] outlined that in qualitative research, the researcher identifies and selects the participants that are knowledgeable about or experienced with a phenomenon of interest. Purposive and convenience sampling were used to select participants. The researchers used their knowledge to purposively sample all women who have experience relating to the women health services at their industries [24]. The justification for the use of convenience sampling was based on the availability and willingness to share their experiences concerning women’s health services at their industries [25]. The sample size was guided by data saturation which was reached at participant number 13.

### 2.4. Data Collection

Data were collected by the research team for one month in December 2019. The existing health services addressing women’s health at the industries were explored through semi-structured one-on-one interviews with an interview guide. The interview guide consisted of logically sequenced questions which were covered with all the participants [24]. Women were asked one central question “*Can you describe the available health services that are specific for women at your industries?*” Probing questions were asked to follow-up on unclear statements [24]. A voice recorder was utilized to capture all interview sessions and field notes were written to capture nonverbal cues. Data was conducted until data saturation was reached in a private room with the interview sessions lasting for about 30–45 min with each participant [24].

### 2.5. Data Analysis

Thematic analysis was used to analyze data by examining narratives from lived experiences from the women in selected industries [18]. Data was transcribed by the principal researcher which was verified by the co-investigators. The researchers generated the initial codes by reading through all the verbatim transcriptions carefully collating data relevant to each code. Relevant topics to the problem were studied then abbreviated as codes and the codes were written next to the appropriate segments of the text. The researchers searched for themes by collating codes into potential themes, all data relevant to each potential theme. Tentative themes were generated by grouping and linking categories to build themes. The themes and sub-themes emerged from this data analysis, the final report of these themes and sub-themes were written in columns [18].

### 2.6. Trustworthiness

Trustworthiness of the project was ensured by applying the following criteria: Credibility was ensured by investing sufficient time in the research field to become familiar with the setting and context, and to get rich information through prolonged engagement with the participants [26]. A thick description of the study context, research methods, and the experiences of the women was done to ensure transferability [24] An audit trail was done by the co-researchers to ensure dependability and confirmability [21].

## 3. Results

Table 3 presents the demographic characteristics of the participants. The interviews were conducted amongst women working in the selected industries until data saturation was reached at participant number 13. The ages of the women in the selected industries ranged from 20 to 58 years. The majority of the participants were married with others being divorced and either single or living with a partner. The majority of the participants were from the cleaning department, while others were safety managers, occupational health nurses and machine operators. Most participants who had more than 1 year working experience and theywere all exposed to different hazards in their departments.

Table 4 below present the summary of findings as themes and sub-themes that emerged from data analysis. The themes and sub-themes emerged from the saturated data during the interviews. 

### 3.1. Diverse Experiences Related to Available Women’s Health Services

The study findings revealed that women have diverse experiences related to the available women’s health services at their industries. The diverseness of women’s experiences is related to the diverse measures of dealing with women’s health, lack of involvement of women in policy/procedures development, and dissatisfaction regarding the current women’s health services provided. The theme is interpreted according to the following sub-themes.

#### 3.1.1. Description of Diverse Measures of Dealing with Women’s Health

Women in industries experience diverse measures for dealing with women’s health. The diverse measures include; general health assessment, screening for breast and cervical cancer, giving of feminine gifts such as pads and wipes, as well as massage vouchers. Furthermore, other participants report that there are no measures to address women’s health issues in the industries, especially those dominated by men. This was confirmed by the following quotes from the participants:

Participant 1: “They give them gifts which in women’s day which include feminine wash and wipes, manicure and massage vouchers, pamphlets on diet and healthy eating, breast self-examination, healthy diets”.

Participant 2: “They are not addressed because most of the staff here are men, we women we are not many and sometimes our supervisors don’t understand when we report things like period pains they think we don’t want to work”.

Participant 3: “And they sometimes teach us on breast cancer and cervical cancer more especially on women’s month”.

#### 3.1.2. Lack of Involvement of Women in Policy/Procedures Development that Affects Women’s Health

The results of the study indicated a concern about women working in the industries regarding their involvement in policies or procedures that relate to women’s health. Participants indicated that there lack of involvement of women in policies/procedures developed which relate to women’s health. The findings are illustrated in the quotes below:

Participant 1: “We are not involved in policy development, usually they involve our unions most of the time”.

Participant 5: “They don’t involve us they just give us policies”.

Participant 12: “They only give us the platform to participate in policies and regulations development through our unions and after that, the unions will inform us on what the agreement was and how we will benefit from the policy, we are not fully engaged on the process”.

#### 3.1.3. Dissatisfaction Regarding the Current Women’s Health Services Provided

The current study findings indicate that women are dissatisfied with the current provided health services in the industries, which neglect most of the specific health needs of women. Women indicated that the industries provide general health services only, and most of the time, its primary healthcare services. The illustration below demonstrates these findings:

Participant 3: *“The is no specific for services women, it is just primary health care which general the only thing which is specific for women is that we supply them with sanitary towel We do give health education on birth control and we encourage them to go the local clinic and do pap smear but this is not enough for a woman”.*

Participant 5: *“They must take our health seriously we have a lot of things sometimes you come to work with stress from home, as women we have a lot of problems which affect our work. They must also allow us to ventilate our problems as women-only thus are important”.*

Participant 9: *“They do not emphasize much on women’s needs e.g., you will find out that you are on your periods and you forgot pads/tampons at home they do not provide us with that which is not good because of the periods are undetectable. They do give us pain medication when we are in pain”.*

The study findings indicate that women have insight regarding women’s health services. Women demonstrated satisfactory knowledge regarding the concept of women’s health and the legislature relevant to address women’s health. The following sub-themes discuss the theme further.

### 3.2. Knowledge Related to Women’s Health Services

#### 3.2.1. Satisfactory Knowledge Regarding the Concept of Women’s Health 

The study findings demonstrated satisfactory knowledge of women working in industries regarding the concept of women’s health. Women were asked to describe their understanding regarding the concept of women’s health, and below are the quotes that indicate their responses.

Participant 6: “Women’s health means you must take care of yourself as women because we are vulnerable to things like cancer. The other thing is we must talk as women to avoid stress because we women have a lot of problems and sometimes we bottle these problems. you can have a stroke”.

Participant 7: “A women’s health is very important as women we must stop having fear and humiliation to go and do different tests that can assist us. Man must respect women by involving men in things like abuse that is affecting and hurting women”.

Participant 10: “Women health relate to issues that concern women then it’s gonna be cancers that affect women, other diseases also, like BP, diabetes it’s gonna be breast cancers and then cervical cancer”.

#### 3.2.2. Satisfactory Knowledge of Basic Employment Act-Maternity Leave

The study findings indicate satisfactory knowledge regarding the basic employment act on services that are relevant to women. For instance, women reported that they are given maternity leave when is due to them. The quotes below support the findings;

Participant 11: “We do have policies which guide us when a female employee pregnant and working in a place where the workload is heavy or not safe for them they are removed and placed in a light-duty and safe place for both mother and baby. They are also entitled to 4 months’ maternity leave and also there is a policy that addresses women’s abuse and sexual abuse were disciplinary procedures take place”.

Participant 12: “I never heard of any policy, but when we are pregnant they give us maternity leave for four months and they also remove us from our stations to light duty”.

Participant 15: “We have policies which address women such as maternity leave policies …they will give you 4 months maternity leave and sometimes they remove you from working inside the plant and put you outside where there is no noise. If you are pregnant you must go to the clinic and inform the sister she will assist you”.

### 3.3. Diverse Description of Women’s Health Services Practice

The study findings demonstrate diverse practices regarding the available women’s health in the industries. The diverse description given by women working in the industries relates to measures taken for women abuse in industries, the health hazards that women are exposed to in industries, and as well as the current practices employed within the industries to promote and maintain women’s health. The detailed description of these measures is presented on the sub-themes below.

#### 3.3.1. Description of Diverse Measures of Addressing Women Violence

Participants indicated diverse measures fr addressing women violence within the industries. The quotes below support the findings;

Participant 5: “They do have policies but I was never affected, sometimes they shout at us but we understand that they want work to be done. They are very strict on violence if you report they discipline them immediately”.

Participant 8: “They take that as a serious offense and they can dismiss you about that”.

Participant 12: “Violence against women is a serious offense and women are encouraged to report where disciplinary measures are taken. If we are abused the union assists us with the case sometimes they suspend them for six months”.

#### 3.3.2. Diverse Health Hazards that Women Are Exposed to

The study findings indicate that women working in the industries are confronted with diverse health hazards. The health hazards such as burns, bumps, injured with bottles, and suffering from inhalation injuries and burns from moving machines, slippery floors, chemicals used for production in the industry, and working in a noisy area. The illustration below demonstrates these findings:

Participant 3: “Moving machinery, noise, and slippery floor, chemicals were they can get burns, lifting of heavy material, moving machinery, forklifts were they can get bumps”.

Participant 2: “standing for a long time, there is also a lot of noise where I work, the bottles can sometimes also injure you”.

Participant 5: “cleaning chemicals that we use can burn you also the inhalations of those chemicals can make you sick. in some departments, the bottles can also cut or injure you”.

#### 3.3.3. Dissatisfaction Versus Satisfaction of the Provided Health Awareness Campaign for Women’s Health

Participants reported dissatisfaction versus satisfaction of the health awareness campaign for women’s health provided within the industries. The quotes below demonstrate these findings.

Participant 6: “we usually have wellness day every year where they call all doctors such as a gynecologist, dentist, optometrist, etc. During this day we bring along our families and they check us and teach us about health issues, but this is not enough for us as women”.

Participant 8: “There is a group that is playing netball and there is a team that organizes awareness for women health were in during women months’ they organize gifts and activities for women. They’re also a team organizing team building for women were in the women go out of province for team building to empower women”.

Participant 9: “In this company, they do wellness campaigns on specific months such as women months were in they teach us on things like cancers and how we check and take of ourselves as women”.

## 4. Discussion

The reproductive health of women at the industries has recently been a major concern with more potential occupational and environmental exposures. Millennium Development Goal 5 aims to improve maternal health through access to good-quality reproductive health care and effective interventions. The current study aimed to explore and describe comprehensive health services that are specific to women’s health at the industries.

The study findings are discussed according to the following three major themes: diverse experiences related to the available women’s health services, knowledge related to women’s health services, and diverse description of women’s health services practice and risks.

### 4.1. Diverse Experiences Related to Available Women’s Health Service

The study findings revealed that women in selected industries experience diverse measures for dealing with women health which embrace the general health assessment, screening for breast and cervical cancer, giving of feminine gifts such as pads and wipes, as well as massage voucher. However, it was specified that most of those measures are only available during special events such as women’s months and wellness day. The industries need to have on-site measures/services necessary to meet the health needs of women on daily basis not depend on special events. Furthermore, industries need to take action to ensure the health and well-being of women at the industries because all employees look at health and safety first and their absence as a demotivator [15,16]. Industries need to put the women’s health services at the heart of efforts to strengthen their health systems to promote access. According to the Work Foundation report, women at the industries have under-recognized chronic gynecological and reproductive health problems that hold back their productivity at work which intern damage their career and earning potential [15]. On the other hand, a study done by Mills and Lin reported that the size of the company can limit the implementation of comprehensive health service plans [27], which could be the factor in the current study as both industries are male-dominated with only 200 females in industry 1 and 160 in industry 2. In support of a study by Wilson on the effect of company size on health and safety, also support that the health and safety implementation plans vary with the size of the company due to the implantation cost which is involved [28]. Another factor could be the issue of the availability of subcontractors in the two selected industries. According to Mills et al. [27,29] mostly the sub-contractors are less well organized with fewer resources to implement a proper OHS system.

The results of the study indicated a concern about women working in the industries regarding their involvement in policies or procedures that relate to women’s health. Participants indicated that there is a lack of involvement of women in policies/procedures developed which relate to women’s health. The European Agency for Safety and Health at Work report [30] documented similar findings wherein women were under-represented in the decision-making concerning occupational health and safety at all levels. They recommended that employers must involve all women directly in the formulation of policies and strategies with more emphasis on women’s opinions, practices, understanding, and expertise. A study was done by Wofford et al. on “A call to action on women’s health” documented that Corporate Social Responsibility (CSR) policy has put more emphasis on shaping corporate policies and practices related to the environment, labor, and human rights, ignoring the health needs of women workers [5].

Employers should improve recognition of women’s reproductive and gynecological health in industries policy and processes. Lewis emphasized the importance of industries/employers to safeguard the culture that permits engagement and discussions about health issues regardless of any gender [31]. The wok foundation 2018 recommended the universal health strategy re-design of policy models for ‘gendered’ health interventions focusing on women and health at the industries inclusive of reproductive and gynecological health conditions and conditions affecting men inclusive of the drug, alcohol abuse, and suicide [5]. A study done by Sommer et al. [32] overlooked issue in low- and middle-income countries and argued that some of the barriers that women are faced within the industries are the none existing or limited guidelines and standards which support menstrual health including women health issues in national occupational health or health and safety regulations.

### 4.2. Knowledge Related to Women’s Health Services

The study findings revealed that women in the industries have insight and knowledge about the importance of women’s health. The study also showed that the industries were informing and implementing the labour relations legislation related to pregnancy and maternity leave. This was displayed by the participants describing the knowledge and practice on maternity leaves and placement during pregnancy in their respective industries. In support of the study found, the South African Board of Women Report documented that in South Africa, once the women report pregnancy at the industries, there are several realities which are done, such as relocation of tasks that might endanger the health of both mother and baby and the provision of four months maternity leave [33].

### 4.3. Diverse Description of Women’s Health Services Practice and Risks

Participants mention the practices concerning the violence against women at the industries which is considered as a serious and dismissible offense. These measures include formulated policies that hold one accountable for his or her actions of violence, appearing in disciplinary hearing and dismissal depending on the hearing outcomes. South African law prohibits any form of sexual harassment or violence in the industries. It is a form of unfair discrimination based on sex, gender, and/or sexual orientation and has been described by the Labour Appeal Court as ’the most heinous misconduct that plagues a industries [34]. The results are in line with the South African Employment Equity Act [35] which guides the employers in the development of policies that hold one accountable for his or her actions of violence, appearing in disciplinary hearing and dismissal depending on the hearing outcomes. Additionally, Botes [36] outlined that the employer must emphasize to their male employees that they will risk their work if they commit any act or commitment of gender-based violence offense while at work. On contrary, it has been documented that in overseas discrimination against women is still a serious challenge particularly sexual harassment and discrimination related to racism and males being superiors [37].

## 5. Ethical Approval and Consent to Participate

The permission to conduct the project was obtained from the University of Limpopo ethics committee (TREC:264/2019: IR). The two selected industries approved the study to be conducted. The participants were informed about the nature of research, the demand it will make on them, and the purpose of the study so that they can make an informed consent to participate. The participants were also be informed about their right to decide to terminate their participation at any stage even if they agreed to participate. Those who agree to participate in the study were to be requested to sign a consent form. The protection of the identity of the participants was ensured by using numbers instead of names during publication.

## 6. Limitations of the Study

Out of five identified food and beverage manufacturing industries in Limpopo Province, only two beverage manufacturing industries gave consent for participation in the study. This has affected the sample size of the study. Despite not having data from other industries, the study is still important even though the results will not be generalized to other industries.

## 7. Conclusions and Recommendations

The industries need to prioritize women’s health more especially that women’s health programs/services in the industries reduce absenteeism, turnover, and increase productivity. The authors conclude that women working in industries are faced with challenges regarding obtaining a comprehensive reproductive women’s health services. For instance, women expressed dissatisfaction in the industries regarding the provided general health and primary healthcare services that have limited women’s health-specific services. Women are exposed to health hazards such as burns, bumps, injuries, and suffering from inhalation injuries and burns from moving machines, noise, slippery floors, and chemicals that are used for production in the industry. Therefore, we conclude that there are no comprehensive health services for women in the selected beverage producing industries. Further studies are recommended to generalize the findings and design comprehensive women health services at beverage producing industries. We, therefore recommend an improvement in the provision of an awareness campaign. The awareness campaign should be offered bi-annual while prioritizing women’s health services such as antenatal services for pregnant women, pap smear test, breast, and cervical cancer screening, counseling services, and supply of feminine products while at the industries. Lastly, we recommend that women should be more involved in the development of policies and procedures that affect women’s health.

## Figures and Tables

**Table 1:** Industry 1: Health Hazard types and classification per department.

Department	Classification of the Hazards	Name of Hazards	Health Problems
1. Syrup room	Chemical	Sugar dust, cleaning chemicals	Allergic rhinitis, irritant, inhalation, allergic dermatitis
	Ergonomics	Standing for a long time and repetitive movements, lifting	Musculoskeletal disorders
	Psychological	Shift work	Poor sleeping and eating habits
	Physical	Noise	Noise induced hearing loss
2. Water treatment	Chemical	Cleaning chemicals	Allergic rhinitis, irritant, inhalation, allergic dermatitis
	Ergonomics	Standing for a long time and repetitive movements, lifting	Musculoskeletal disorders
	Psychological	Shift work	Poor sleeping and eating habits
	Physical	Slippery floor, noise	Strains and sprains, noise-induced hearing loss
3. Laboratory	Chemical	Laboratory chemicals	Allergic rhinitis, irritant, inhalation, allergic dermatitis
	Ergonomics	Sitting/standing for a long time	Musculoskeletal disorders
4. Packaging	Chemical	Cleaning chemicals	Allergic rhinitis, irritant, inhalation, allergic dermatitis
	Ergonomics	Standing for a long time and repetitive movements, lifting	Musculoskeletal disorders
	Psychological	Shift work	Poor sleeping and eating habits
	Physical	Noise, moving machinery, fork lift	Noise-induced hearing loss, accidents, bums
5. Ware house	Chemical	Cleaning chemicals	Allergic rhinitis, irritant, inhalation, allergic dermatitis
	Ergonomics	Standing for a long time and repetitive movements	Musculoskeletal disorders
	Psychological	Shift work	Poor sleeping and eating habits
	Physical	Noise	Noise induced hearing loss
6. Stores	Chemical	Cleaning chemicals	Allergic rhinitis, irritant, inhalation, allergic dermatitis
	Ergonomics	Standing for a long time and repetitive movements	Musculoskeletal disorders
	Psychological	Shift work	Poor sleeping and eating habits
7. Cooler	Chemical	Cleaning chemicals	Allergic rhinitis, irritant, inhalation, allergic dermatitis
	Ergonomics	Standing for a long time and repetitive movements	Musculoskeletal disorders
	Psychological	Shift work	Poor sleeping and eating habits
	Physical	Cold	Hypothermia Susceptibility to colds and flu
8. Engineering	Chemical	Cleaning chemicals	Allergic rhinitis, irritant, inhalation, allergic dermatitis
	Ergonomics	Standing for a long time and repetitive movements, lifting	Musculoskeletal disorders
	Psychological	Shift work	Poor sleeping and eating habits
	Physical	Noise, heat (boiler), dust (coal dust boiler)	Noise-induced hearing loss, fatigue, exhaustion, susceptibility to cold and flu
9. Canteen	Chemical	Cleaning chemicals	Allergic rhinitis, irritant, inhalation, allergic dermatitis
	Ergonomics	Standing for a long time and repetitive movements	Musculoskeletal disorders
	Psychological	Shift work	Poor sleeping and eating habits
	Physical	Heat	Burns
10. Waste sorting	Chemical	Cleaning chemicals	Allergic rhinitis, irritant, inhalation, allergic dermatitis
	Ergonomics	Standing for a long time and repetitive movements, lifting	Musculoskeletal disorders
	Psychological	Shift work	Poor sleeping and eating habits
	Physical	Slippery floor, noise	Strains and sprains, noise-induced hearing loss
	Ergonomics	Poor ergonomics inactivity	

**Table 2:** Industry 2: Health Hazard types and classification per department.

Department	Classification of the Hazards	Name of Hazards	Health Problems
1. Brewing	Chemical	Cleaning chemicals	Irritant, inhalation, allergic dermatitis
	Ergonomics	Standing for a long time and repetitive movements, lifting	Musculoskeletal disorders
	Psychological	Shift work	Poor sleeping and eating habits
	Physical	Noise	Noise induced hearing loss
2. Depot sales	Chemical	Cleaning chemicals	Allergic rhinitis, irritant, inhalation, allergic dermatitis
	Ergonomics	Standing for a long time and repetitive movements, lifting	Musculoskeletal disorders
	Psychological	Shift work	Poor sleeping and eating habits
	Physical	Noise	Noise induced hearing loss
3. Quality Laboratory	Chemical	Laboratory chemicals	Allergic rhinitis, irritant, inhalation, allergic dermatitis
	Ergonomics	Sitting/standing for a long time	Musculoskeletal disorders
4. Packaging	Chemical	Cleaning chemicals	Allergic rhinitis Irritant, inhalation, allergic dermatitis
	Ergonomics	Standing for a long time and repetitive movements, lifting	Musculoskeletal disorders
	Psychological	Shift work	Poor sleeping and eating habits
	Physical	Noise, moving machinery, forklift	Noise-induced hearing loss, accidents, bums
5. Warehouse	Chemical	Cleaning chemicals	Allergic rhinitis, irritant, inhalation, allergic dermatitis
	Ergonomics	Standing for a long time and repetitive movements	Musculoskeletal disorders
	Psychological	Shift work	Poor sleeping and eating habits
	Physical	Noise	Noise induced hearing loss
6. Stores	Chemical	Cleaning chemicals	Allergic rhinitis, irritant, inhalation, allergic dermatitis
	Ergonomics	Standing for a long time and repetitive movements	Musculoskeletal disorders
	Psychological	Shift work	Poor sleeping and eating habits
7. Engine room	Chemical	Cleaning chemicals	Allergic rhinitis, irritant, inhalation, allergic dermatitis
	Ergonomics	Standing for a long time and repetitive movements	Musculoskeletal disorders
	Psychological	Shift work	Poor sleeping and eating habits
	Physical	Cold	Hypothermia, susceptibility to colds and flu
8. Depot	Chemical	Cleaning chemicals	Allergic rhinitis, irritant, inhalation, allergic dermatitis
	Ergonomics	Standing for a long time and repetitive movements, lifting	Musculoskeletal disorders
	Psychological	Shift work	Poor sleeping and eating habits
	Physical	Noise, heat (boiler), dust (coal dust boiler)	Noise induced hearing loss Fatigue, exhaustion, susceptibility to cold and flu
9. Canteen	Chemical	Cleaning chemicals	Allergic rhinitis, irritant, inhalation, allergic dermatitis
	Ergonomics	Standing for a long time and repetitive movements	Musculoskeletal disorders
	Psychological	Shift work	Poor sleeping and eating habits
	Physical	Heat	Burns
	Ergonomics	Standing for a long time and repetitive movements, lifting	Musculoskeletal disorders
	Psychological	Shift work	Poor sleeping and eating habits
	Physical	Slippery floor, noise	Strains and sprains, noise-induced hearing loss
	Ergonomics	Poor ergonomics inactivity	

**Table 3:** Demographic characteristics of women in two beverage producing industries.

Gender	Age	Marital Status	Occupation	Years of Experience	Hazards Classification
Females (13)	20–28 (5) 29–38 (4)	Single (6) Living with partner (1)	Safety manager (1) Cleaning manager (2) Occupational health nurse (2)	Under 1 year (1) 1–5 (4) 6–10 (4)	Chemicals (cleaning chemicals) Physical (noise, slippery floors, lifting, trapping)
	39–48 (2) 49–58 (2)	Married (5) Divorced (1)	Machine operator (3) Cleaner (5)	11–15 (3) Over 20 years (1)	Mechanical (moving machinery and bottles)

**Table 4:** Summary of the themes and sub-themes reflecting the experiences of women related to comprehensive health services for women at the industries.

Themes	Sub-Themes
1. Diverse experiences related to the available women’s health services	Description of the diverse measures in dealing with women’s health Lack of involvement of women in women’s health policy/procedures development that affects women Explanation of dissatisfaction regarding the current women health services provided
2. Knowledge related to women’s health services	Satisfactory knowledge regarding the concept of women’s health Description of existing knowledge related to basic employment act on different leave provision Existence of limited knowledge regarding women-specific health services
3. Description of practices and risks related to women’s health services	Description of diverse measures of addressing women violence Existence of health hazards that women are exposed to Dissatisfaction versus satisfaction of the existing health awareness campaign for women’s health
